# A 3’-UTR Polymorphism in Soluble Epoxide Hydrolase Gene Is Associated with Acute Rejection in Renal Transplant Recipients

**DOI:** 10.1371/journal.pone.0133563

**Published:** 2015-07-31

**Authors:** Guillermo Gervasini, Montserrat García-Cerrada, Eliecer Coto, Esther Vergara, Guadalupe García-Pino, Raul Alvarado, Maria Jesús Fernández-Cavada, Beatriz Suárez-Álvarez, Sergio Barroso, Emilio Doblaré, Carmen Díaz-Corte, Carlos López-Larrea, Juan Jose Cubero

**Affiliations:** 1 Department of Medical and Surgical Therapeutics, Division of Pharmacology, Medical School, University of Extremadura, Badajoz, Spain; 2 Molecular Genetics, Laboratory of Medicine, Hospital Universitario Central de Asturias (HUCA), Oviedo, Spain; 3 Service of Immunology, Infanta Cristina University Hospital, Badajoz, Spain; 4 Service of Nephrology, Infanta Cristina University Hospital, Badajoz, Spain; 5 Laboratory of Molecular Biology of Renal Disease, Health Research Institute F. Jimenez-Diaz, Universidad Autónoma, Madrid, Spain; 6 Service of Nephrology, HUCA, Oviedo, Spain; 7 Service of Immunology, HUCA, Oviedo, Spain; 8 Red de Investigación Renal, Instituto Salud Carlos III, Madrid, Spain; University of Florida, UNITED STATES

## Abstract

**Background and Purpose:**

Epoxyeicosatrienoic acids (EETs) are arachidonic acid metabolites that play a protective role against damaging processes that may occur after re-oxygenation of the graft. We aimed to investigate whether the presence of functional polymorphisms in the gene encoding soluble epoxy hydrolase (*EPHX2*), which metabolizes EETs to less active compounds, may play a role in the outcome of renal transplantation.

**Methods:**

In a group of 259 Caucasian renal transplant recipients and 183 deceased donors, we determined the presence of three common *EPHX2* SNPs, namely rs41507953 (K55R), rs751141 (R287Q) and rs1042032 A/G. Associations with parameters of graft function and the incidence of acute rejection were retrospectively investigated throughout the first year after grafting by logistic regression adjusting for clinical and demographic variables.

**Results:**

Carriers of the rs1042032 GG genotype displayed significantly lower estimated glomerular filtration rate (eGFR) (38.15 ± 15.57 vs. 45.99 ± 16.05; p = 0.04) and higher serum creatinine values (1.57 ± 0.58 vs. 1.30 ± 0.47 g/dL; p=0.02) one year after grafting, compared to patients carrying the wildtype A-allele. The same GG genotype was also associated to increased risk of acute rejection. Interestingly, this association was observed for the genotype of both recipients [OR =6.34 (1.35-29.90); p = 0.015] and donors [OR = 5.53 (1.10-27.80); p=0.042]. A statistical model including both genotypes along with other meaningful demographic and clinical variables resulted in an increased significance for the association with the recipients’ genotype [OR=8.28 (1.21-74.27); p=0.031].

**Conclusions:**

Our results suggest that genetic variability in the EETs-metabolizing gene, *EPHX2*, may have a significant impact on the outcome of deceased-donor renal transplantation.

## Introduction

The arachidonic acid (AA) is metabolized by cytochrome P450 (CYP) enzymes to a number of compounds with important biological functions. The epoxygenase branch of this pathway leads to the synthesis of epoxyeicosatrienoic acids (EETs), which are considered to be endothelium-derived hyperpolarizing factors that regulate the intracellular transport of electrolytes and maintain vascular smooth muscle tone with vasodilator and anti-inflammatory properties [1–3]. In turn, EETs are metabolized to less active dihydroxyeicosatrienoic acids (DHETs) by soluble epoxide hydrolase (sEH) [4].

There presently is an increasing body of evidence suggesting that these EETs may have a significant function in organ transplantation. Thus, these compounds have been shown to play a protective role in the kidney [5,6], particularly against damaging processes that may occur after re-oxygenation of the graft [7–9]. Indeed, increasing the levels of EETs, via sEH inhibition, has been proposed as a therapeutic strategy in renal diseases [2].

The EET-metabolizing enzyme sEH is encoded by the *EPHX2* gene, which is expressed in the kidney and presents single nucleotide polymorphisms (SNPs) that have been associated with altered enzyme activity [10,11]. Previous disease-association studies have shown that certain genetic variations in *EPHX2* are related to the risk of coronary heart disease and ischemic stroke [12,13]. In addition, one recent study in rodents has reported that sEH activity determines the severity of ischemia-reperfusion injury in the kidney [14]. To our knowledge, only two studies by the same research group have analyzed the impact of *EPHX2* genetic variability on kidney disease and transplantation. The authors showed that some of these variants can affect the progression of human IgA nephropathy and may be predictive of allograft dysfunction, but no assessment was made on their impact on acute rejection [15,16].

With this background, we hypothesize that the presence of polymorphisms with an established functional and/or clinical relevance in the *EPHX2* gene, namely rs41507953 (K55R), rs751141 (R287Q) and rs1042032 in the 3’ untranslated region (UTR), may play a role in the outcome of renal transplantation. To test this hypothesis, we retrospectively analyzed these variants in a population of renal transplant recipients and donors and searched for associations with graft dysfunction and acute rejection episodes (ARE).

## Subjects and Methods

From an initial number of 354 clinical records reviewed, a total of 259 adult renal transplant recipients were included in the final study sample (patients with incomplete records of clinical parameters, demographic characteristics or those with failed genotyping were ruled out from the study). Part of these patients had already been studied in previous works by our group [17–19]. The patients were all of Caucasian origin and received a single kidney at two Spanish centers, the Infanta Cristina Hospital in Badajoz and the Hospital Universitario Central de Asturias in Oviedo. All transplants were carried out with deceased donors from whom genetic material was available in 183 cases.

After the transplant, a triple immunosuppressive therapy was implemented with mycophenolate mofetil (2 g/day), a tapering schedule of corticoids (500 mg IV methylprednisolone at the time of surgery, 125 mg intravenously (IV) the following day and then 20 mg of oral prednisone daily, progressively tapered to 5 mg daily at 2 months after transplantation) and either cyclosporine or tacrolimus. Tacrolimus starting dose was set to 0.1 mg/kg administered twice a day. Initial dosage of Cyclosporine was 4–10 mg/kg/day divided into two administrations. The first dose was administered orally shortly before transplantation or IV in the perioperative period when the patient's condition did not support the enteral route. The amount of drug administered IV was one third of the oral dose. Further doses of immunosupressants were subsequently adjusted according to blood concentrations. Tacrolimus and cyclosporine blood concentrations were routinely measured using an immunoassay performed on a Cobas Mira Plus analyzer (Roche Diagnostics).

Acute allograft rejection was established by histological findings in renal biopsies according to the Banff classification and/or by clinical evaluation as previously described [18,20]. Delayed graft function (DGF) was defined as the need for dialysis within the first week after transplantation. DGF and ARE data were retrospectively retrieved from clinical records up until the first year after grafting. To estimate death-censored allograft survival, patients were followed up until the earliest of graft loss (defined as the absence of kidney function, occurring any time after transplantation due to irreversible graft injury requiring chronic dialysis and/or re-transplantation), death with a functioning graft or December 31, 2013.

Renal function was assessed by estimating the glomerular filtration rate (eGFR) from serum creatinine using the Modification of Diet in Renal Disease (MDRD) formula [21]: eGFR (ml min^-1^ 1.73 m^-2^) = 186 x (Serum Creatinin^-1.154^ x Age^-0.203^) x (1.212 if Black) x (0.742 if Female). Several studies have reported that among renal transplanted patients the MDRD values show a better correlation with the true filtration rate in comparison with other renal function estimates, e.g. raw serum creatinine or the Cockcroft-Gault formula [22].

### Ethics Statement

All participants gave oral and written consent for their participation. The study was approved by the Ethics Committee of the Infanta Cristina Hospital (Reference No. 18002657), and was conducted in accordance with the Declaration of Helsinki and its subsequent revisions.

### Genotype analysis

Genomic DNA was isolated by using a QIAamp DNA Blood Kit (Qiagen, Hilden, Germany) from either whole blood samples, in the case of the recipients, or from previously frozen lymphocytes obtained from donors. All three *EPHX2* SNPs (Table 1) were identified by real-time PCR using TaqMan SNP Genotype Assays from Life Technologies (Rockville, MD, USA).

**Table 1:** Location, consequence and context sequence of the three *EPHX2* SNPs analyzed in this study, as provided by the manufacturer of the TaqMan SNP genotyping assays (Life Technologies, Maryland, USA).

SNP	Transition	Aminoacid change	Location	Context sequence [VIC/FAM]
rs41507953	A/G	K55R	Chr.8: 27358505 on NCBI Build 37	GAGGGTGCCACTACCCGGCTTATGA[A/G]AGGAGAGATCACACTTTCCCAGGTG
rs751141	A/G	R287Q	Chr.8: 27373865 on NCBI Build 37	CCTGCTCTGGCCCAGGCAGGTTACC[A/G]GGTCCTAGCTATGGACATGAAAGGC
rs1042032	A/G	None (3’-UTR)	Chr.8: 27402074 on NCBI Build 37	TGTGCCCACGCTCAGCAGGTGTGCC[A/G]TCCTTCCACCTGCTGGGGCACCATT

UTR, untranslated region.

For the haplotype study, the SNPstats platform [23] was utilized to provide linkage disequilibrium data and to estimate the effect of haplotypes on the risk for acute rejection by linear regression modelling; regression parameters pertained to the log odds ratios adjusted by the same clinical and demographic variables used in the single-SNP study (see below). Frequency threshold for rare haplotypes was set at 0.01.

### Statistical analyses

Fisher’s exact or Pearson’s X^2^ test were used for the univariate analysis of the associations between categorical data (i.e. genotypes vs. clinical events). In order to compare quantitative variables (e.g. eGFR) between the different genotype groups, T-student or ANOVA tests were used depending on the number of groups considered. Multivariate regression analysis was performed to collectively assess the impact of both genetic and non-genetic parameters. Analyses were adjusted for demographic and clinical covariates according to statistical significance in univariate studies and/or clinical criteria described elsewhere [18,24]. Variables included were age of donors and recipients, type of immunosuppression, presence of DGF, cold ischemia time > 24 hours, number of HLA mismatches ≥ 3, and peak cytotoxic PRA ≥50%. The age of both donors and recipients was transformed into categorical variables using the median values as cut-off points (49 and 51 years for recipients and donors, respectively). Association of the three *EPHX2* SNPs with death-censored allograft survival was analyzed by Cox regression analyses.

SNPstats was used to determine the adequate model of inheritance (additive, dominant or recessive). Statistical analysis was performed using the Statistical Package for the Social Sciences (SPSS) version 15.0 for Windows (SPSS Inc., Chicago, Ill. USA). In all instances differences were considered to be significant when p values were lower than 0.05.

## Results

Demographic and clinical characteristics of the 259 transplant recipients analyzed in the study are described in Table 2. The most common primary kidney disease was glomerulonephritis (42.1%), followed by chronic interstitial nephritis (11.2%) and polycystic kidney disease (8.7%). Several other conditions accounted for 14.2% of cases. In 23.8% of the patients the specific condition could not be determined. Induction therapy was implemented in 53 subjects (20.5%) with antibodies against interleukin-2 receptor (basiliximab) and in 9 patients (3.5%) with thymoglobulin.

**Table 2:** Clinical and demographic parameters of the study population. Four time-points within a one-year follow-up were considered.

Time-point		One week	One month	5 months	One year
Creatinine serum concentration (mg/dL)		2.48 ± 2.07	1.69 ± 0.93	1.49 ± 0.72	1.38 ± 0.62
eGFR (ml min^-1^ 1.73 m^-2^)		33.29 ± 14.10	36.90 ± 13.73	41.60 ± 13.98	44.02 ± 14.83
Recipients on tacrolimus	208 (80.3)				
Dose (mg/kg)		0.14 ± 0.07	0.11 ± 0.06	0.08 ± 0.04	0.06 ± 0.06
Dose-normalized Tac blood concentration (ng/ml per mg/day per kg)		111.0 ± 80.1	121.1 ± 66.7	162.5± 77.0	176.3 ± 110.1
Recipients on cyclosporine	51 (19.7)				
Dose (mg/kg)		8.0 ± 1.9	6.3 ± 1.6	4.5 ±1.7	3.5 ± 1.1
Dose-normalized CsA blood concentration (ng/ml per mg/day per kg)		42.32 ± 20.13	53.91 ± 22.48	51.04 ± 17.96	50.16 ± 18.98
Recipients sex (male/female)	158 (61.0) / 101 (39.0)				
Recipients age (years)	48.18 ± 14.31				
Type of dialysis (hemodialysis/peritoneal)	174 (67.2)/85 (32.8)				
Duration of dialysis before transplantation (months)	38.16 ± 30.09				
Donor age (years)	47.63 ± 17.51				
Number of transplants (first/second/third)	246 (95.0)/11 (4.2)/1 (0.8)				
Cold ischemia time (hours)	16.22 ± 5.01				
Peak cytotoxic PRA ≥ 50%	20 (7.72)				
HLA mismatch					
0–2	69 (26.64)				
3–4	162 (62.54)				
5–6	28 (10.81)				

Data are shown as number (percentage) or mean ± standard deviation.

Genotype and minor allele frequencies, both in donors and recipients, are shown in Table 3. Allele frequencies in the population of study were all in Hardy-Weinberg equilibrium (p-values > 0.05).

**Table 3:** Genotypic and allelic frequencies in donors and renal transplant recipients.

		Recipients		Donors		
Polymorphism		N	%	N	%	MAF
*EPHX2 K55R*, rs41507953	*KK*	215	83.0	133	72.7	0.114
	*KR*	40	15.4	47	25.7	
	*RR*	4	1.5	3	1.6	
*EPHX2 R287Q*, rs751141	*RR*	223	86.1	153	83.6	0.076
	*RQ*	36	13.9	29	15.8	
	*QQ*	0	0.0	1	0.5	
*EPHX2 3’UTR A>G*, rs1042032	*AA*	153	59.1	81	44.3	0.273
	*AG*	85	32.8	90	49.2	
	*GG*	21	8.1	12	6.6	

N, number of subjects; MAF, minor allele frequency.

### Association of genetic polymorphisms in donors and recipients with graft function and survival

**Fig 1:**
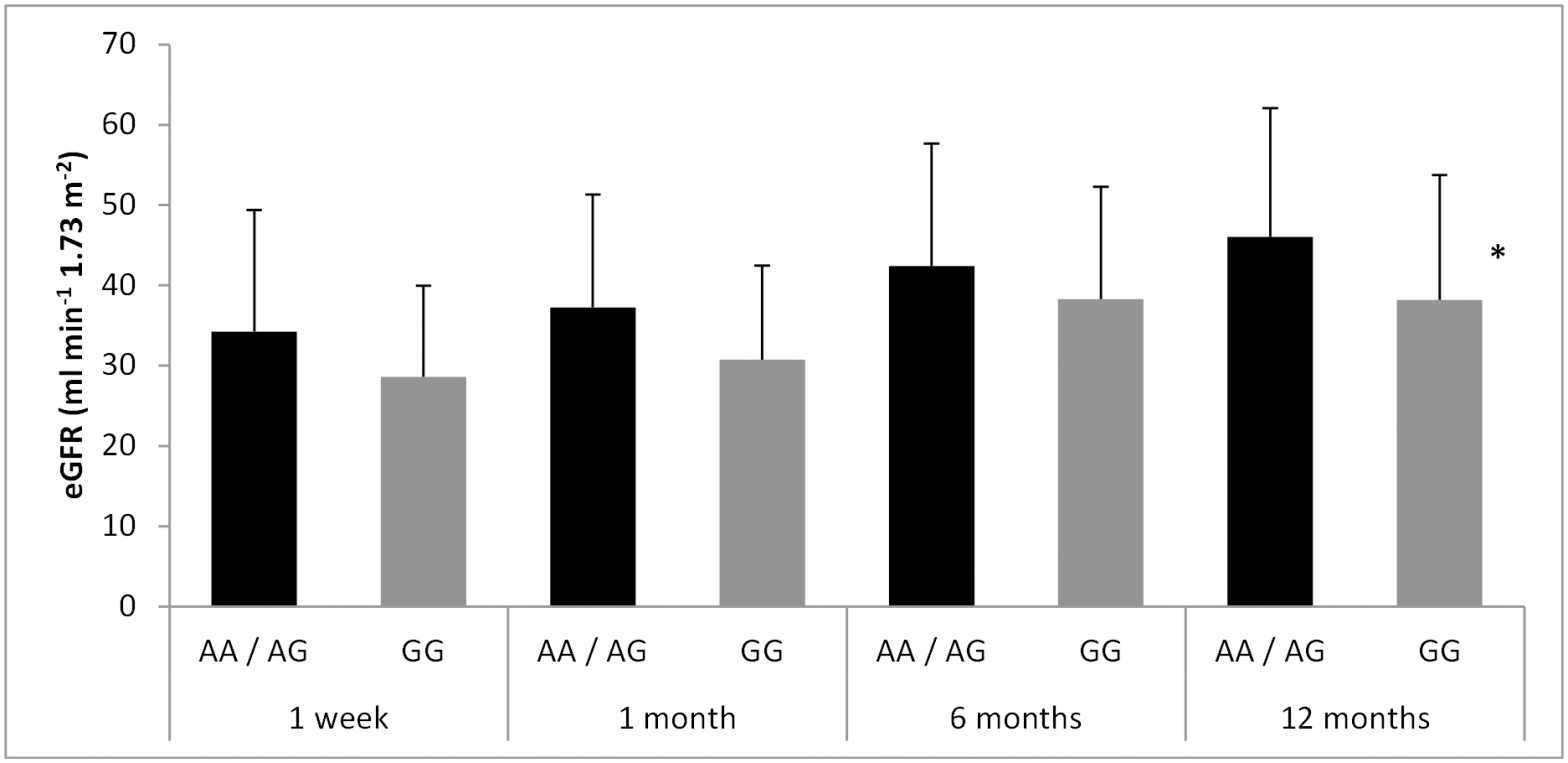
Effect of the *EPHX2* 3’ UTR A/G polymorphism (rs1042032) of the recipient on the estimated glomerular filtration rate throughout the one-year follow-up. *p<0.05 vs. AA/AG group.

**Fig 2:**
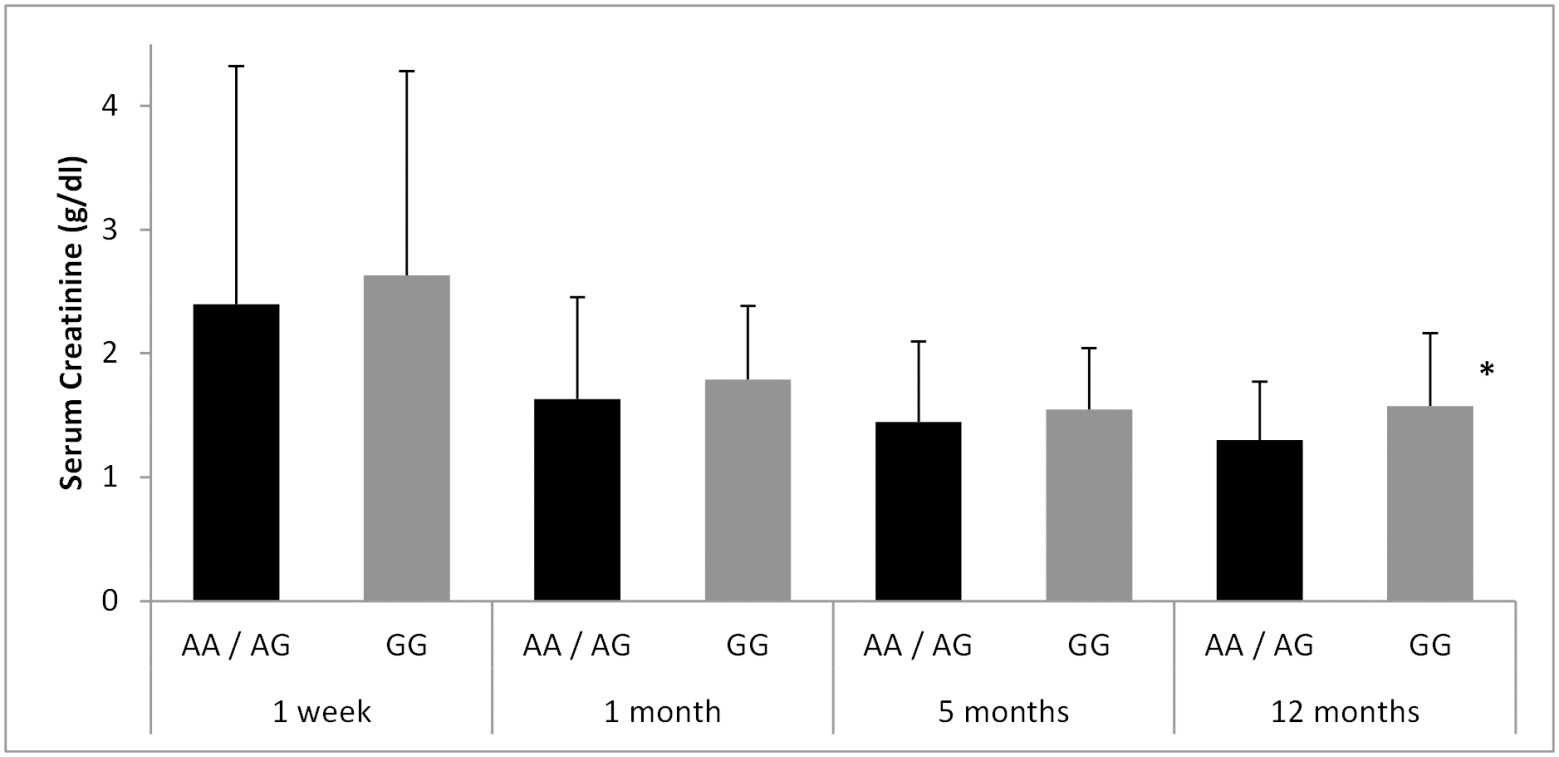
Effect of the *EPHX2* 3’ UTR A/G polymorphism (rs1042032) of the recipient on serum creatinine concentrations throughout the one-year follow-up. *p<0.05 vs. AA/AG group.

The eGFR in the recipients increased from 33.29 ± 14.07 to 44.02 ± 14.83 in the one-year follow-up period (p = 9.3 e-22). Amongst the SNPs analyzed, we found that subjects who carried the rs1042032 GG genotype displayed worse estimated renal function at all four time-points considered, compared to carriers of the wild type A-allele (Fig 1). Differences reached statistical significance at the end of the one-year follow-up (38.15 ± 15.57 vs. 45.99 ± 16.05 for GG and AA/AG respectively; p = 0.04). In a similar manner, the same genotype was also associated to higher serum creatinine values throughout the study (Fig 2) and again the difference was significant twelve months after grafting (1.57 ± 0.58 vs. 1.30 ± 0.47 g/dL for GG and AA/AG respectively; p = 0.02). In contrast, neither the rs1042032 GG genotype in the donor nor the other two SNPs analyzed showed a relevant effect on the measured renal function (S1 and S2 Tables show mean eGFR and serum creatinine values for the rs41507953 and rs751141 polymorphisms).

The mean follow-up time for the graft survival study was 114.65 ± 60.07 months. There were no differences with regard to death-censored allograft survival when patients were stratified according to their genotype or that of their donors. Hazard ratios with 95% confidence intervals obtained by Cox regression analyses are shown in S3 Table.

### Association of genetic polymorphisms in donors and recipients with acute rejection

Thirty-one patients (11.96%) experienced ARE according to the diagnostic criteria described in Methods. The incidence of acute rejection was significantly more frequent in carriers of the rs1042032 GG genotype, compared to subjects with the wildtype AA genotype (Table 4). In addition, a borderline association was observed for the GG genotype of the donor [OR = 4.80 (0.96–26.54)]. When the participants were analyzed using a recessive model (rs1042032 GG vs. AA/AG) the significance level of the association further increased [OR = 6.34 (1.35–29.90), p = 0.015 and OR = 5.53 (1.10–27.80), p = 0.042 for recipient and donor genotypes, respectively]. In the case of the recipients’ genotype, the association remained significant after correction for multiple testing.

**Table 4:** Odds ratios (OR) with 95% confidence intervals (CI) for the association of *EPHX2* SNPs with acute rejection in renal transplant recipients.

		Recipients		Donors	
Polymorphism		OR (CI)	p	OR (CI)	p
*EPHX2 K55R*	*KK*	Ref.	0.83	Ref.	0.43
	*KR*	1.23 (0.35–4.28)		1.53 (0.49–4.79)	
	*RR*	NC		6.61 (0.28–156.51)	
*EPHX2 R287Q*	*RR*	Ref.	0.38	Ref.	0.48
	*RQ*	1.93 (0.46–8.01)		1.77 (0.35–7.24)	
	*QQ*	NC		NC	
*EPHX2 3’UTR A>G*	*AA*	Ref.	0.04	Ref.	0.11
	*AG*	0.64 (0.15–2.67)		0.76 (0.25–2.33)	
	*GG*	5.45 (1.09–27.21)		4.80 (0.96–26.54)	

NC, non-calculable.

In order to determine the relative weight of the donor vs. recipient rs1042032 GG genotype in the risk for ARE, we created a statistical model that included both genotypes along with significant demographic and clinical covariates described in methods. The results shows that the significance of the donor rs1042032 GG genotype decreased slightly [OR = 5.84 (0.82–37.88), p = 0.078]; in contrast, the OR value for the recipient’s GG genotype increased up to an OR of 8.28 (1.21–74.27) with a p-value of 0.031. This genotype presented the highest regression coefficient of all variables tested (B = 2.11). Amongst non-genetic factors, the impact of DGF, which was present in 29.1% of the patients and had shown a strong association with ARE in univariate analysis [OR = 9.66 (3.68–25.35); p < 0.001] decreased to a borderline association [OR = 2.72 (0.94–11.66)]. DGF occurrence was not significantly associated with any of the studied SNPs. The model explained 36.3% of the variability in the sample.

Finally, we tested whether there was any modification of ARE risk when the three *EPHX2* loci were analyzed combined. Linkage disequilibrium data (D’, r^2^) were 0.99, 0.008 for rs41507953/rs751141; 0.78, 0.186 for rs41507953/rs1042032 and 0.92, 0.196 for rs751141/ rs1042032 in recipients and 0.99, 0.016; 0.75, 0.109 and 0.95, 0.307 for the same SNP pairs in donors. Table 5 shows the different haplotypes identified and their association with the risk for ARE. Haplotype distribution was not different between donors and recipients (Chi-square p > 0.05). Only haplotype *2 in the recipient (rs41507953 A / rs751141 A/ rs1042032 G) displayed a statistical trend towards higher risk of ARE [OR = 2.28 (0.92–5.67), p = 0.08]. The analyses were adjusted by the same variables as in the single-SNP study.

**Table 5:** Effect of *EPHX2* haplotypes both in donors and recipients on the risk for acute rejection.

Haplotype	Frequency	rs41507953	rs751141	rs1042032	OR (CI)	p
Recipients						
*1	0.732	wt	wt	wt	Reference	
*2	0.114	wt	wt	M	2.28 (0.92–5.67)	0.08
*3	0.082	M	wt	M	0.24 (0.01–4.14)	0.33
*4	0.065	wt	M	M	3.09 (0.76–12.52)	0.12
*5	<0.01	M	wt	wt	-	
Donors						
*1	0.662	wt	wt	wt	Reference	
*2	0.116	wt	wt	M	0.94 (0.26–3.37)	0.93
*3	0.077	M	wt	M	1.96 (0.53–7.29)	0.32
*4	0.137	wt	M	M	2.21 (0.81–6.00)	0.12
*5	<0.01	M	wt	wt	-	

wt, wild type allele. M, variant allele.

## Discussion

Some ten years ago, two studies revealed that a *CYP3A5* allele was able to predict tacrolimus concentration-to-dose ratios in renal transplant recipients [25,26]. This finding prompted the publication of a great number of reports aimed to determine an association between genetic variants and meaningful clinical parameters in renal transplantation. The vast majority of these studies have focused on the variability in the *CYP3A* and *ABCB1* genes, which are responsible for the metabolism and transport of anticalcineurin inhibitors [27,28]. However, it is somewhat surprising the lack of attention that has attracted the role of *EPHX2* variants in the field of renal transplantation. It is even more so when EETs, the substrates for the *EPHX2*-encoded sEH, are vital components of the renal and vascular response to injury such as that produced in ischemia-reperfusion [2,29]. Indeed, AR9281 and GSK2256294 are new sEH inhibitors that are being tested in clinical trials to assess their efficacy and safety for the treatment of hypertension and other cardiovascular diseases [30,31].

In the present paper we have evaluated the impact of common genetic variability in the gene coding for sEH, the enzyme responsible for EETs degradation in the kidney. We observed that the GG genotype of the rs1042032 A/G SNP was associated with lower eGFR and higher serum creatinine values, particularly late in the study period. Only one previous study by Lee et al. [16] has analyzed the clinical role of this SNP in renal transplantation. The authors did not find a relevant impact of the GG genotype on their patients’ renal function, quite the opposite; they reported an association of the wild type AA genotype with allograft dysfunction. In contrast with recent evidence (see below) this study assumed that the SNP resulted in reduced enzyme activity, although the authors failed to confirm this in vitro. Some facts could also explain the discrepancy with our results. First, the patients in the study by Lee et al. were Asian who displayed a much higher MAF for the SNP (0.445 vs. 0.273 in our patients). Second, their study setting was living-donor transplantation, which generally means lower rates of graft dysfunction and acute rejection [32]; and last but not least, the study by Lee et al. only detected an association of the AA genotype with worse renal function when participants were stratified into two groups (High/Low eGFR), because when raw numbers were considered eGFR values were not significantly different. In fact, it is interesting that, in line with our results, serum creatinine values were higher in carriers of the variant G-allele [16]. In any case, our findings should be interpreted cautiously, given the existence of contradictory results. On the other hand, we did not observe a significant effect of the rs1042032 SNP on long-term graft survival. It is tempting to speculate that, in the long term, the protective effect of EETs [2,14] may be eclipsed by other factors such as chronic exposure to medication or the occurrence of infections.

The most relevant finding of this study was the fact that the rs1042032 GG genotype, both in donors and recipients, was associated with a higher risk of ARE after adjusting for demographic and clinical covariates. Interestingly, the only two recipients that carried the GG genotype and also received a kidney from a GG donor experienced ARE. The most likely explanation for this observation must be an increase of enzymatic activity produced by the SNP, because that would imply a faster rate of EETs degradation, subsequent endothelial dysfunction, increased blood pressure and, eventually, glomerular injury, as it has previously suggested [2]. In line with this hypothesis, two recent studies have demonstrated that enhanced activity of sEH increases the severity of the ischemia-reperfusion injury in the kidney [14] and is also associated with more advanced endothelial dysfunction [33], which are common complications in kidney transplantation [34]. Moreover, recent data available in the Genevar (GENe Expression VARiation) database [35] at the Sanger Institute website (http://www.sanger.ac.uk/resources/software/genevar) seem to confirm that this SNP is associated with increased enzyme activity. Genevar charts show that subjects from different ethnicities carrying the rs1042032 GG genotype, which we found to be related to increased risk of acute rejection and worse renal function, display significantly higher expression of sEH (S1 Fig). This would presumably lead to lower levels of EETs and, consequently, to a lower endogenous capacity of counteracting damaging processes in the graft. Furthermore, an increased sEH activity could also be the explanation for the borderline association with ARE observed with haplotype *2 in the recipient, since this allele combination contained the aforementioned 1042032 G variant and two wild type alleles in the other loci; an allele constellation that would presumably lead to a faster rate of EETs degradation.

The statistical model proposed in the present work indicates that the *EPHX2* genotype of the recipient was more important to predict ARE than that of the donor, which underlines the importance of vascular and/or inflammatory mechanisms at a systemic level. In line with this finding, it was also the 1042032 GG genotype of the recipient, but not that of the donor, that was associated with worse graft function. Indeed, along with kidney EETs concentrations, decreased levels of vascular EETs have also been associated with renal diseases [2]. Future studies including circulating levels of EETs and, particularly, the actual expression of these compounds in the graft may help elucidate this question.

In any case, the statistical model presented herein should be validated in larger cohorts and, as such, has more of an academic than a clinical value. In this regard, a limitation of this work was its relatively small sample size, which may have been particularly relevant for some wide, although significant, confidence intervals. In addition, availability of genetic material from deceased donors was incomplete, which was not unexpected as DNA was obtained from lymphocytes that in some cases had been frozen for years. Another limitation was the lack of data on circulating levels of EETs (a parameter not available in a retrospective study) or on enzyme expression in the graft, which could have been helpful to confirm the proposed hypotheses. In this regard, protocol biopsies in subjects with stable graft function or in those with unambiguous clinical data are not performed in our centers, and therefore there was not enough tissue available as to carry out protein expression studies in this series of patients. This lack of histological confirmation for all acute rejection episodes may have also affected the reported incidence of this complication. On the other hand, the fact that the donor genotype was available greatly increased the significance of some findings, such as the aforementioned association with acute rejection observed for the rs1042032 SNP of both donors and recipients.

In conclusion, in a study designed with mechanism-linked, established polymorphisms, we have shown for the first time to our knowledge, that genetic variability in the EETs-metabolizing gene, *EPHX2*, may have a significant impact on the outcome of deceased-donor renal transplantation. The explanation for this finding most likely implies the modulation of the levels of EETs, with vasoactive and anti-inflammatories properties. The findings described in the present work may be the first step in a new field of research on the role of genetic variants affecting EETs availability in renal transplant. Further work with larger, independent series of patients and/or additional genes involved in EETs synthesis is warranted to confirm the findings described herein.

## Supporting Information

S1 Fig *EPHX2* rs1042032 association with soluble epoxy hydrolase expression in eight HapMap3 populations. Spearman's rho, nominal p-value and permutation p-value are shown above each plot (https://www.sanger.ac.uk/resources/software/genevar).
(TIF)

S1 Table Effect of the *EPHX2* K55R (rs41507953) and R287Q (rs751141) polymorphisms of both donors and recipients on the estimated glomerular filtration rate (eGFR) throughout the one-year follow-up. Mean and standard deviation (SD) values are shown. *Only one donor carried the 287QQ genotype.
(DOCX)

S2 Table Effect of the *EPHX2* K55R (rs41507953) and R287Q (rs751141) polymorphisms of both donors and recipients on serum creatinine concentrations throughout the one-year follow-up. Mean and standard deviation (SD) values are shown. *Only one donor carried the 287QQ genotype.
(DOCX)

S3 Table Cox regression analysis of death-censored graft survival according to *EPHX2* genotypes. Hazard ratio values were adjusted by donor age, acute rejection, delayed graft function, type of immunosuppression and PRA peak. HR, hazard ratio; CI, 95% confidence intervals. ^a^Individuals were grouped in AA vs. AG+GG to make it up for the low number of GG carriers. ^b^There were no recipients with the GG genotype. ^c^In order to keep consistency with the other analyses performed in the study, the recessive model of inheritance is shown for the rs1042032 SNP. ^d^Only one donor carried the GG genotype and was ruled out from the analysis.
(DOCX)
