# Supplementary figures and images for: A 3’-UTR Polymorphism in Soluble Epoxide Hydrolase Gene Is Associated with Acute Rejection in Renal Transplant Recipients
Source: PLoS One. 2015 Jul 31;10(7):e0133563. doi: 10.1371/journal.pone.0133563 (PMC4521874; doi:10.1371/journal.pone.0133563)

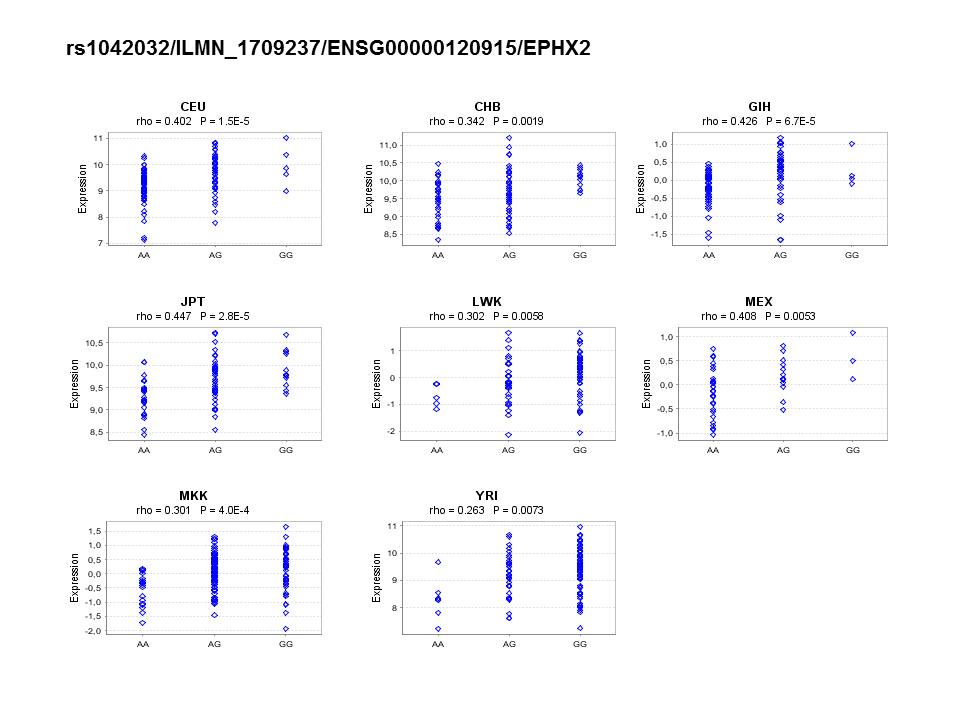

Supplement: S1 Fig — Spearman's rho, nominal p-value and permutation p-value are shown above each plot (https://www.sanger.ac.uk/resources/software/genevar). (TIF) [file pone.0133563.s001.tif]
