# Supplementary material for: A 3’-UTR Polymorphism in Soluble Epoxide Hydrolase Gene Is Associated with Acute Rejection in Renal Transplant Recipients
Source: PLoS One. 2015 Jul 31;10(7):e0133563. doi: 10.1371/journal.pone.0133563 (PMC4521874; doi:10.1371/journal.pone.0133563)
Supplement: S1 Table — Mean and standard deviation (SD) values are shown. *Only one donor carried the 287QQ genotype. (DOCX) [file pone.0133563.s002.docx]

**S1 Table.** Effect of the *EPHX2* K55R (rs41507953) and R287Q (rs751141) polymorphisms of both donors and recipients on the estimated glomerular filtration rate (eGFR) throughout the one-year follow-up. Mean and standard deviation (SD) values are shown.

|  |  |  | **eGFR** | | | |
| --- | --- | --- | --- | --- | --- | --- |
| **Recipients** |  |  | *1 week* | *1 month* | *6 months* | *12 months* |
| *EPHX2 K55R* | KK | Mean | 31.98 | 34.16 | 40.78 | 44.09 |
|  |  | SD | 13.62 | 14.56 | 15.28 | 17.59 |
|  | KR | Mean | 33.88 | 30.64 | 40.87 | 44.53 |
|  |  | SD | 20.07 | 13.56 | 22.64 | 22.21 |
|  | RR | Mean | 25.49 | 34.34 | 43.21 | 44.74 |
|  |  | SD | 1.72 | 6.38 | 3.64 | 10.14 |
| *EPHX2 R287Q* | RR | Mean | 31.79 | 33.63 | 41.32 | 45.45 |
|  |  | SD | 15.43 | 14.69 | 16.96 | 18.89 |
|  | RQ | Mean | 36.95 | 36.02 | 42.39 | 40.46 |
|  |  | SD | 7.89 | 12.94 | 16.65 | 13.94 |
| **Donors** |  |  |  |  |  |  |
| *EPHX2 K55R* | KK | Mean | 31.76 | 33.68 | 40.01 | 42.26 |
|  |  | SD | 15.65 | 15.61 | 17.43 | 17.83 |
|  | KR | Mean | 31.39 | 36.17 | 42.28 | 47.78 |
|  |  | SD | 13.11 | 15.28 | 15.92 | 19.87 |
|  | RR | Mean | 29.01 | 34.98 | 40.77 | 41.57 |
|  |  | SD | 2.33 | 4.98 | 2.98 | 7.02 |
| *EPHX2 R287Q* | RR | Mean | 32.79 | 34.63 | 40.68 | 44.44 |
|  |  | SD | 14.77 | 14.72 | 15.83 | 19.13 |
|  | RQ | Mean | 25.95 | 33.39 | 40.52 | 40.49 |
|  |  | SD | 15.10 | 18.92 | 22.37 | 14.18 |
|  | QQ | Mean | 12.00 | 18.00 | 42.00 | 53.00 |
|  |  | SD^*^ | - | - | - |  |

*Only one donor carried the 287QQ genotype
