# Supplementary material for: A 3’-UTR Polymorphism in Soluble Epoxide Hydrolase Gene Is Associated with Acute Rejection in Renal Transplant Recipients
Source: PLoS One. 2015 Jul 31;10(7):e0133563. doi: 10.1371/journal.pone.0133563 (PMC4521874; doi:10.1371/journal.pone.0133563)
Supplement: S2 Table — Mean and standard deviation (SD) values are shown. *Only one donor carried the 287QQ genotype. (DOCX) [file pone.0133563.s003.docx]

**S2** **Table.** Effect of the *EPHX2* K55R (rs41507953) and R287Q (rs751141) polymorphisms of both donors and recipients on serum creatinine concentrations throughout the one-year follow-up. Mean and standard deviation (SD) values are shown.

|  |  |  | **Serum creatinine (g/dL)** | | | |
| --- | --- | --- | --- | --- | --- | --- |
| **Recipients** |  |  | *1 week* | *1 month* | *6 months* | *12 months* |
| *EPHX2 K55R* | KK | Mean | 2.47 | 1.69 | 1.49 | 1.35 |
|  |  | SD | 1.99 | 0.96 | 0.64 | 0.52 |
|  | KR | Mean | 3.03 | 1.64 | 1.57 | 1.45 |
|  |  | SD | 2.43 | 0.61 | 0.84 | 0.48 |
|  | RR | Mean | 2.21 | 1.33 | 1.12 | 1.01 |
|  |  | SD | 0.23 | 0.18 | 0.16 | 0.08 |
| *EPHX2 R287Q* | RR | Mean | 2.50 | 1.69 | 1.49 | 1.34 |
|  |  | SD | 2.04 | 0.95 | 0.70 | 0.52 |
|  | RQ | Mean | 2.81 | 1.51 | 1.46 | 1.34 |
|  |  | SD | 2.34 | 0.67 | 0.54 | 0.46 |
| **Donors** |  |  |  |  |  |  |
| *EPHX2 K55R* | KK | Mean | 2.48 | 1.73 | 1.56 | 1.41 |
|  |  | SD | 2.00 | 0.98 | 0.73 | 0.57 |
|  | KR | Mean | 2.86 | 1.77 | 1.60 | 1.45 |
|  |  | SD | 2.37 | 0.87 | 0.67 | 0.62 |
|  | RR | Mean | 2.66 | 1.88 | 1.74 | 1.55 |
|  |  | SD | 0.32 | 0.23 | 0.28 | 0.65 |
| *EPHX2 R287Q* | RR | Mean | 2.55 | 1.72 | 1.55 | 1.41 |
|  |  | SD | 2.13 | 0.95 | 0.67 | 0.55 |
|  | RQ | Mean | 2.46 | 1.78 | 1.73 | 1.49 |
|  |  | SD | 1.67 | 0.73 | 0.94 | 0.76 |
|  | QQ | Mean | 7.00 | 4.50 | 1.20 | 1.00 |
|  |  | SD^*^ | - | - | - | - |

*Only one donor carried the 287QQ genotype
