# Supplementary material for: A 3’-UTR Polymorphism in Soluble Epoxide Hydrolase Gene Is Associated with Acute Rejection in Renal Transplant Recipients
Source: PLoS One. 2015 Jul 31;10(7):e0133563. doi: 10.1371/journal.pone.0133563 (PMC4521874; doi:10.1371/journal.pone.0133563)
Supplement: S3 Table — Hazard ratio values were adjusted by donor age, acute rejection, delayed graft function, type of immunosuppression and PRA peak. HR, hazard ratio; CI, 95% confidence intervals. aIndividuals were grouped in AA vs. AG+GG to make it up for the low number of GG carriers. bThere were no recipients with the GG genotype. cIn order to keep consistency with the other analyses performed in the study, the recessive model of inheritance is shown for the rs1042032 SNP. dOnly one donor carried the GG genotype and was ruled out from the analysis. (DOCX) [file pone.0133563.s004.docx]

**S3 Table.**

Cox regression analysis of death-censored graft survival according to *EPHX2* genotypes. Hazard ratio values were adjusted by donor age, acute rejection, delayed graft function, type of immunosuppression and PRA peak.

| **SNP** | **HR (CI)** | **p** |
| --- | --- | --- |
| Recipient rs41507953^a^ (K55R) |  |  |
| AA | 0.44 (0.05-3.81) | 0.455 |
| AG/GG |  |  |
| Recipient rs751141^b^ (R287Q) |  |  |
| AA | 1.72 (0.34-8.66) | 0.508 |
| AG |  |  |
| Recipient rs1042032^c^ |  |  |
| AA/AG | 0.48 (0.05-4.52) | 0.520 |
| GG |  |  |
| Donor rs41507953^a^ (K55R) |  |  |
| AA | 1.33 (0.45-3.95) | 0.611 |
| AG/GG |  |  |
| Donor rs751141^d^ (R287Q) |  |  |
| AA | 0.37 (0.05-2.84) | 0.339 |
| AG |  |  |
| Donor rs1042032^c^ |  |  |
| AA/AG | 1.98 (0.61-6.41) | 0.256 |
| GG |  |  |

HR, hazard ratio; CI, 95% confidence intervals

^a^Individuals were grouped in AA vs. AG+GG to make it up for the low number of GG carriers.

^b^There were no recipients with the GG genotype

^c^In order to keep consistency with the other analyses performed in the study, the recessive model of inheritance is shown for the rs1042032 SNP

^d^Only one donor carried the GG genotype and was ruled out from the analysis
